# *Mycobacterium leprae* and *Mycobacterium lepromatosis* in small mammals in Midwest Brazil

**DOI:** 10.1016/j.bjid.2024.103874

**Published:** 2024-09-24

**Authors:** Beatriz Silva Nogueira, Maerle Oliveira Maia, Ravena Fernanda Braga de Mendonça, Luciano Nakazato, Valéria Dutra

**Affiliations:** a Universidade Federal de Mato Grosso, Faculdade de Medicina Veterinária, Departamento de Microbiologia e Biologia Molecular, Cuiabá, MT, Brazil; b Universidade Federal de Mato Grosso, Instituto de Biociências, Departamento de Biologia e Zoologia, Cuiabá, MT, Brazil

**Keywords:** RLEP, RLPM, Amazonia, Cerrado

## Abstract

Leprosy is a chronic infectious disease caused by the bacilli *Mycobacterium leprae* and *Mycobacterium lepromatosis*. In addition to humans, animals such as nine-banded armadillos and red squirrels are species naturally infected. The objective of this study was to investigate the presence of *M. leprae* and *M. lepromatosis* in non-volant small mammals of the order Didelphimorphia and Rodentia through Polymerase Chain Reaction (PCR) assay. During 2015 and 2018, field expeditions were carried out in three municipalities, covering biotic elements of the Amazon and Cerrado biomes, in the Mato Grosso State, Midwest of Brazil. A specific primer for repetitive sequences of the genomic DNA of *M. leprae* and *M. lepromatosis* targeting the RLEP and RLPM gene, respectively, was used to screen for these agents. The molecular detection of *M. leprae* DNA in the samples was 13.8%. *M. lepromatosis* was not detected. The present study reports a description of *M. leprae* in small non-volant mammals in Brazil.

Leprosy, also known as hanseniasis or Hansen's disease, is an infectious disease caused by the bacterial pathogens *Mycobacterium leprae* and *Mycobacterium lepromatosis*.^1^ It occurs mainly in tropical and subtropical countries and is considered a neglected tropical disease.^2^

Although *M. leprae* is considered a human-only pathogen, evidence of zoonotic cases has emerged after exposure to nine-banded armadillos (*Dasypus novemcinctus*) in the Americas.^3^ The transmission mechanisms between animals and humans are not well established; however, the probable route is aerosol transmission.^4^ Some studies have also reported the survival of this microorganism in the environment, amoebas and arthropods, which may contribute to the maintenance of the disease.^5^^,^^6^

As for *M. lepromatosis*, the global extent of infection and its effect on the development of hanseniasis are still unknown.^7^ This pathogen has already been reported in humans in Brazil, Mexico, the United States, and Southeast Asia.^8^ Recent studies have indicated that both *M. leprae* and *M. lepromatosis* may be involved in the development of leprosy.^9^

Countless questions about leprosy remain regarding its transmission and ecology, as well as its zoonotic and sapronotic reservoirs. Thus, the objective of this study was to investigate the presence of *M. leprae* and *M. lepromatosis* infections in non-volant small mammals of the orders Didelphimorphia and Rodentia in Brazil.

During 2014 and 2018, field expeditions were conducted in the municipalities of Alta Floresta (09°58′S, 56°04′W) and Sinop (11°49′S, 55°24′W), encompassing biotic elements from the Amazon, and Barra do Garças (15°54′S, 52°16′W), encompassing the Cerrado biome in Mato Grosso State in Midwestern Brazil. Small non-volant mammals were captured using wire cage traps and Sherman-like traps. The animals were sampled primarily for another project investigating the epidemiology of tick-borne diseases in small mammals^10^^,^^11^ and the stored samples were made available for the present study. The captured mammals were anesthetized by intramuscular injection of ketamine hydrochloride/xylazine solution. All captured mammals were euthanized by increasing the anesthetic dose.

DNA extraction from 10 mg of spleen tissue was performed and PCR was used to screen the *M. leprae* using specific primers (R1 5′-CGC CCG GAT CCT CGA TGC AC-3 ' and R2 5′-GCA CGT AAG CTT GTC GGT GG-3′) targeting a fragment of a 372-base pair repetitive sequence corresponding to the RLEP gene region.^12^ The expected amplicon sizes were purified and prepared for sanger sequencing. The obtained sequences were then queried using the Basic Local Alignment Search Tool (BLAST) to determine the closest identities with congeneric organisms available in GenBank. For the detection of *M. lepromatosis*, samples positive for the RLEP region were prepared using the TaqMan Universal PCR Master Mix (Applied Biosystems) with a TaqMan probe (5′-AAGTGACGCGGGCGTGGATT-3′) and specific primers (5′-TTGGTGATCGGGGTCGGCTGGA-3′; 5′-CCCCACCGGACACCACCAACC-3′) to amplify the RLPM region.^13^

Procedures in this study were approved by the Ethics Committee on Animal Research of the Federal University of Mato Grosso (CEUA protocol n° 23108.076870/2015-41) and “Instituto Chico Mendes de Conservação da Biodiversidade” (ICMBio permit n° 8863-1).

A total of 269 small non-volant mammals were surveyed for the presence of *M. leprae*. Overall, *M. leprae* was detected in 37 (13.8%) of 269 spleen samples, as evaluated using the RLEP PCR assay. Table 1 shows the detection of *M. leprae* in the small mammals. The DNA of a specimen of *Didelphis albiventris, Cerradomys* sp., *Neacomys* sp. and *Thrichomys pachyurus* was sequenced, obtaining from 99% to 100% of identity to the corresponding sequences of *M. leprae* (MF975706.1, MF975705.1) available on GenBank (Table 2). In the Amazon biome, three animals (1.1%) were positive for *M. leprae* infection, while in the Cerrado biome the positivity rate was 43.0% (n = 34). *M. lepromatosis* was not detected in any of the samples tested using the RLPM RT-PCR assay.

**Table 1:** Detection of *Mycobacterium leprae* in samples of marsupials and rodents in the state of Mato Grosso, Brazil.

**Municipalities**	**Mammal species (n)**	***Mycobacterium leprae* detected**
		**N° tested/N° positive (%)**
Alta Floresta	**Order *didelphimorphia***	
	*Caluromys philander* (1)	1/0 (0)
	*Cryptonanus* sp. (9)	9/1 (11.1)
	*Didelphis marsupialis* (2)	2/0 (0)
	*Glironia venusta* (1)	1/0 (0)
	*Marmosa constantiae* (9)	9/1 (11.1)
	*Marmosa murina* (1)	1/0 (0)
	*Marmosops bishop* (7)	7/0 (0)
	*Marmosops aff. pinheroi* (3)	3/0 (0)
	*Monodelphis glirine* (3)	3/0 (0)
	*Monodelphis saci* (2)	2/0 (0)
	**Order *rodentia***	
	*Hylaeamys megacephalu* (3)	3/0 (0)
	*Neacomys amoenus* (5)	5/0 (0)
	*Necromys Lasiurus* (2)	2/0 (0)
	*Oecomys cleberi* (2)	2/0 (0)
	*Oecomys paricola* (2)	2/0 (0)
	*Oecomys aff. catherinae* (1)	1/0 (0)
	*Oligoryzomys cf* (2)	2/0 (0)
	Proechimys sp. (4)	4/0 (0)
Sinop	**Order *didelphimorphia***	
	*Caluromys philander* (5)	5/0 (0)
	*Didelphis marsupials* (28)	28/0 (0)
	*Gracilinanus peruanus* (3)	3/0 (0)
	*Marmosa constantiae* (49)	49/0 (0)
	*Marmosa murina* (3)	3/0 (0)
	*Metachirus nudicaudatus* (4)	4/0 (0)
	**Order *rodentia***	
	*Mesomys hispidus* (3)	3/0 (0)
	*Mus musculus* (1)	1/0 (0)
	*Oecomys bicolor* (19)	19/0 (0)
	*Oecomys paricola* (1)	1/0 (0)
	*Oecomys roberti* (4)	4/0 (0)
	*Proechimys roberti* (11)	11/1 (9.0)
Barra do Garças	**Order *didelphimorphia***	
	*Didelphis albiventris* (6)	6/3 (50)
	*Gracilinanus agilis* (1)	1/1 (100)
	*Monodelphis domestica* (1)	1/0 (0)
	**Order *rodentia***	
	*Cerradomys* sp. (3)	3/1 (33.3)
	*Neacomys* sp. (1)	1/1 (100)
	*Oecomys* sp. (2)	2/0 (0)
	*Thrichomys pachyurus* (65)	65/28 (43.1)

**Table 2:** Samples from rodents and marsupials positive for *Mycobacterium leprae* sent for sequencing.

**Municipality**	**Species (n° of samples)**	**GenBank Homology (%)**
Barra do Garças	**Order *didelphimorphia***	
	*Didelphis albiventris* (1)	271/273pb (99)
	**Order *rodentia***	
	*Cerradomys* sp. (1)	247/249pb (99)
	*Neacomys* sp. (1)	238/238pb (100)
	*Thrichomys pachyurus* (1)	242/242pb (100)

This study is one of the few to report the presence of *M. leprae* infection in small non-volant mammals in Brazil. The organ of choice was the spleen because previous studies with experimentally infected armadillos have demonstrated the spleen with high rates of recovery from bacilli, with yields about 4 to 10 times higher than that of the liver.^14^

The state of Mato Grosso is a historic leprosy endemic area and the sustained occurrence of leprosy patients at hyperendemic levels (> 65/100,000 inhabitants)^15^ in most municipalities of the state may, in part, be associated with operational improvements in health services, including better coverage and decentralization.

The Cerrado (savanna) biome has been a global biodiversity hotspot with high rates of native vegetation suppression and wildfires over the past three decades. The samples collected in the Cerrado biome were located in the Serra Azul State Park, created in 1994 aiming at its environmental conservation. The area was previously occupied by indigenous groups and after colonization there was an intense occupation by miners, demonstrating an ancient anthropic impact in the region.^16^ Currently visitation to the Park is constant by residents and tourists. Considering that a person with multibacillary leprosy eliminates an estimate of 107 bacilli per day through nasal secretions,^17^ the survival of the agent in the environment and the high rates of leprosy in the state of Mato Grosso, the historical anthropic impact in the region that remains can favor the maintenance of the microorganism in the environment and consequent infection of the individuals who live there.

Worldwide, the detection of animals infected with *M. leprae* is still low; however, several groups have reported the possibility of reservoirs in wildlife, including in non-human primates, margay (*Leopardus wiedii*), and lowland tapirs (*Tapirus terrestris*),^18^ armadillos^3^ and red squirrels (*Sciurus vulgaris*).^19^ Thus, a role for animals in the persistence and transmission of *M. leprae* is increasingly cited as a real possibility.^8^

No *M. lepromatosis* was detected in any of the tested samples. Although studies have reported the presence of this agent in red squirrels in Europe,^19^ there is little research on its presence in leprosy-endemic countries. In a study by Schilling et al.,^20^ *M. lepromatosis* was not detected in rodent samples from Mexico, which is similar to the findings of the present study.

The discovery of *M. leprae* in small non-volant mammals and its potential relationship with high leprosy rates in Mato Grosso, Brazil, is intriguing; however, it is important to note that to accurately estimate the risk presented by non-human reservoirs in transmitting this disease, more research is needed to identify additional leprosy reservoirs. This knowledge is crucial for developing better strategies for controlling the spread of these microorganisms in the future.

## Ethics approval

Procedures in this study were previously approved by the Ethics Committee on Animal Research of the Federal University of Mato Grosso (CEUA protocol n° 23108.076870/2015-41) and “Instituto Chico Mendes de Conservação da Biodiversidade” (ICMBio permit n° 8863-1). All legal requirements and guidelines in Brazil for the care and use of animals have been followed.

## Conflicts of interest

The authors declare that they have no known competing financial interests or personal relationships that could have appeared to influence the work reported in this paper.

## References

[1] Han X.Y., Seo Y.H., Sizer K.C. (2008). A new Mycobacterium species causing diffuse lepromatous leprosy. Am J Clin Pathol.

[2] Hambridge T., Nanjan Chandran S.L., Geluk A., Saunderson P., Richardus J.H (2021). Mycobacterium leprae transmission characteristics during the declining stages of leprosy incidence: a systematic review. PLoS Negl Trop Dis.

[3] Ploemacher T., Faber W.R., Menke H., Rutten V., Pieters T. (2020). Reservoirs and transmission routes of leprosy; a systematic review. PLoS Negl Trop Dis.

[4] Honap T.P., Pfister L.A., Housman G. (2018). Mycobacterium leprae genomes from naturally infected nonhuman primates. PLoS Negl Trop Dis.

[5] Holanda MV de, Marques L.E.C., Macedo MLB de (2017). Presence of Mycobacterium leprae genotype 4 in environmental waters in Northeast Brazil. Rev Soc Bras Med Trop.

[6] Ferreira J da S, Souza Oliveira D.A., Santos J.P. (2018). Ticks as potential vectors of Mycobacterium leprae: use of tick cell lines to culture the bacilli and generate transgenic strains. PLoS Negl Trop Dis.

[7] Cardona-Castro N., Escobar-Builes M.V., Serrano-Coll H., Adams L.B., Lahiri R. (2022). Mycobacterium lepromatosis as cause of leprosy, Colombia. Emerg Infect Dis.

[8] Deps P., Antunes JMA de P, Collin S.M (2021). Zoonotic risk of Hansen's disease from community contact with wild armadillos: a systematic review and meta-analysis. Zoonoses Public Health.

[9] Fernández J.D.P., Pou-Soarez V.E., Arenas R. (2022). Mycobacterium leprae and & Mycobacterium lepromatosis infection: a report of six multibacillary cases of leprosy in the Dominican Republic. Jpn J Infect Dis.

[10] Colle A.C., Mendonça R.F.B., Maia MO (2020). Rickettsial survey and ticks infesting small mammals from the Amazon forest in midwestern Brazil. Syst Appl Acarol.

[11] Pacheco T dos A., Muñoz-Leal S., Maia MO (2021). Molecular detection of Rickettsia spp. in ticks associated with non-volant small mammals from the Brazilian Cerrado, with notes on a divergent morphotype of Ornithodoros Guaporensis. Int J Acarol.

[12] Woods S.A., Cole S.T (1990). A family of dispersed repeats in Mycobacterium leprae. Mol Microbiol.

[13] Sharma R., Singh P., McCoy R.C. (2020). Isolation of Mycobacterium lepromatosis and development of molecular diagnostic assays to distinguish Mycobacterium leprae and M. lepromatosis. Clin Infect Dis.

[14] da Silva M.B., Portela J.M., Li W. (2018). Evidence of zoonotic leprosy in Pará, Brazilian Amazon, and risks associated with human contact or consumption of armadillos. PLoS Negl Trop Dis.

[15] Brasil. Ministério da Saúde. Secretaria de Vigilância em Saúde e Ambiente. Boletim Epidemiológico Hanseníase 2024. Avaiable at: https://www.gov.br/saude/pt-br/centrais-de-conteudo/publicacoes/boletins/epidemiologicos/especiais/2024/be_hansen-2024_19jan_final.pdf/view

[16] Fundação Estadual do Meio Ambiente (FEMA-MT). Governo do Estado de Mato Grosso. Coordenadoria de Unidades de Conservação. Plano de Manejo do Parque Estadual da Serra Azul. vol. 3, 2022. Available at: http://sema.mt.gov.br/site/index.php/unidades-de-conservacao/unidades-de-conserva%C3%A7%C3%A3o-estaduais/category/174-parque-estadual-da-serra-azul

[17] Davey T.F., Rees R.J.W. (1974). The nasal discharge in leprosy: clinical and bacteriological aspects. Lepr Rev.

[18] Maruyama F.H., Morgado T.O., Pacheco R.C., Nakazato L., Dutra V. (2018). Molecular detection of Mycobacterium leprae by Polymerase chain reaction in captive and free-ranging wild animals. Braz J Infect Dis.

[19] Avanzi C., del-Pozo J., Benjak A. (2016). Red squirrels in the British Isles are infected with leprosy bacilli. Science.

[20] Schilling A.K., Avanzi C., Ulrich R.G. (2019). British red squirrels remain the only known wild rodent host for leprosy bacilli. Front Vet Sci.

